# Effects of oral *Nigella sativa* oil on the expression levels and serum concentrations of adiponectin, PPAR-γ, and TNF-α in overweight and obese women: a study protocol for a crossover-designed, double-blind, placebo-controlled randomized clinical trial

**DOI:** 10.1186/s13063-019-3568-0

**Published:** 2019-08-17

**Authors:** Elham Razmpoosh, Sara Safi, Mahta Mazaheri, Amin Salehi-Abargouei, Nooshin Abdollahi, Majid Nazari, Hossein Fallahzadeh, Azadeh Nadjarzadeh

**Affiliations:** 1 Department of Nutrition, Faculty of Health, Shahid Sadoughi University of Medical Sciences, Yazd, Iran; 2 Nutrition and Food Security Research Center, Shahid Sadoughi University of Medical Sciences, Yazd, Iran; 3 Faculty of Medicine, Shahid Sadoughi University of Medical Sciences, Yazd, Iran; 4 Department of Medical Genetics, Shahid Sadoughi University of Medical Sciences, Yazd, Iran; 5 Department of Biostatistics and Epidemiology, Faculty of Health, Shahid Sadoughi University of Medical Sciences, Yazd, Iran

**Keywords:** Obesity, Overweight, *Nigella sativa*, Tumor necrosis factor-alpha, Adiponectin, Peroxisome proliferator-activated receptors

## Abstract

**Background:**

Obesity is a major public health problem in recent decades. The accumulation of excessive fat promotes inflammatory status. Meanwhile, herbal products are marketed for their weight-loss properties, such as *Nigella sativa* (*N. Sativa*) which has been used for centuries to treat rheumatoid arthritis, diabetes, and asthma; recently, the anti-obesity characteristics of *N. sativa* have also been indicated. However, the exact mechanisms and cellular-related pathways are still unclear. Thus, we will aim to assess the effects of oral *N. sativa* on the gene expression of inflammatory and adipogenesis-related factors, including TNF-α, PPAR-γ, and adiponectin as well as assessing their serum concentrations among obese and overweight individuals.

**Methods:**

Obese and overweight women aged 25–55 years with a body mass index (BMI) of 25–35 kg/m^2^ will be recruited from the Obesity Clinic in Shahid Sadoughi University of Medical Sciences and will be assessed for eligibility against inclusion criteria. They will be randomly assigned into two groups to receive either two capsules of *N. sativa* or two capsules of placebo per day for eight weeks (each capsule contains 1000 mg of *N. sativa* or placebo). There will be a four-week wash-out period and then participants will receive the reverse supplements for another eight weeks. Biochemical assessments and gene expressions (using real-time polymerase chain reaction) will be conducted at the beginning and at the end of every intervention period.

**Discussion:**

The present study will investigate the probable cellular pathways for the anti-obesity effects of *N. sativa* in overweight/obese women.

**Trial registration:**

Iranian Registry of Clinical Trials, IRCT20180528039884N1. Registered on 2nd of July, 2018.

**Electronic supplementary material:**

The online version of this article (10.1186/s13063-019-3568-0) contains supplementary material, which is available to authorized users.

## Background

The epidemic of overweight and obesity presents a major challenge to health around the world. Obesity is defined as the accumulation of abnormal or excessive fat that may interfere with the maintenance of an optimal state of health [1]. The excess of macronutrients in the adipose tissues stimulates them to release inflammatory mediators such as tumor necrosis factor alpha (TNF-α) and reduces production of adiponectin, predisposing to a pro-inflammatory state and oxidative stress [2].

Among the adipokines, adiponectin is widely recognized for its anti-diabetic, anti-inflammatory, anti-atherogenic, and cardio-protective effects and shows protective activity in various processes such as energy metabolism and inflammation [3]. In humans, plasma levels of adiponectin are decreased in cases of insulin resistance and obesity [4]. One of the molecular mechanisms of adiponectin may be direct actions via downregulating of inflammatory responses involving TNF-α [5]. In obesity, alterations in the gene expression of adiponectin and its receptors reduce adiponectin sensitivity leading to insulin resistance [3].

On the other hand, peroxisome proliferator-activated receptors (PPARs) are a group of nuclear receptors with various isoforms, including α, β/δ, and γ, that are involved in transcription regulation of a broad range of genes related to inflammation and energy homeostasis and represent important targets for obesity, obesity-induced inflammation, and metabolic syndrome. PPAR-γ is a transcription factor abundant in adipose tissue which has been known to regulate adipocyte differentiation and fatty acid storage [6]. More recently, PPAR-γ has been recognized as playing an important role in inhibiting the expression of inflammatory cytokine [7]. Obesity has been reported to induce a decline in the activity and amount of PPAR-γ. This correlation appears to be strongly associated with the pathogenesis of obesity [8]. An association between adiponectin and PPAR-γ has been reported by Yadav et al.; they revealed that adiponectin can mediate phosphorylation of adenosine monophosphate-activated protein kinase and also PPARs, after binding to its receptors and thereby increase fatty acid oxidation [9]. An animal study on rats reported that increased adiponectin levels and improvements in insulin sensitivity after a weight-reduction intervention, might be due to an increase in PPAR-γ levels [10].

With regard to the relation between TNF-α and obesity, plasma concentration of TNF-α and its receptors were found to be higher in obese individuals, which may get alleviated with weight loss [11]. Interestingly, TNF-α is reported to have a correlation with PPAR-γ activity. Studies revealed that TNF-α might have inhibitory effects on PPAR-γ which is involved in the pathogenesis of inflammation [12, 13]. Furthermore, a previous study reported that PPAR-γ activity could be regulated by TNF-α at pre-translational and post-translational levels [13]. More recently, the long-term assessment of anti-TNF-α inhibitor treatment to individuals diagnosed with metabolic syndrome has been shown to increase adiponectin levels, confirming a role for TNF-α in obesity-related insulin resistance in humans [12]. Moreover, TNF-α inhibits the conversion of pre-adipocytes to mature adipocytes, notably through downregulating adipogenic genes such as PPAR-γ, allowing further recruitment of uncommitted cells and, thus, possible expansion of adipose tissue mass [14]. TNF-α also downregulates the messenger RNA (mRNA) levels of adiponectin [15] while, conversely, adiponectin suppresses lipopolysaccharide-induced TNF-α production [5].

Studies have shown that losing weight in obese individuals can result in an increase in adiponectin levels and the expression of its receptors [3, 15]. Meanwhile, herbal products are widely marketed for their weight-loss properties [16]. *Nigella sativa* (*N. sativa*) is a traditional herbal medicine that has been used for centuries to treat rheumatoid arthritis, diabetes, asthma, and other metabolic disorders. Recently, anti-obesity characteristics of *N. sativa* have also been indicated [17]. The exact mechanism of anti-obesity effects of *N. sativa* is unclear; bioactive components of *N. sativa* including Thymoquinone, inhibitory components of lipase, unsaturated fatty acids, and its appetite-reducing effect are involved in probable mechanisms for anti-obesity effects of *N. sativa* [18]. Additionally, *N. sativa* has suggested to act as an agonist of PPAR-γ which impacts upon energy homeostasis, thus enhancing the expression of lipogenic genes [19]. Concerning the effects of *N. sativa* on gene expression, there are only in vitro, in vivo, and/or animal trials that evaluated the effects *N. sativa* or its major active component, Thymoquinone, on the gene expression of various factors such as TNF-α, adiponectin, or PPAR-γ, and reported beneficial improvements in inflammation [20–27]. Nevertheless, the probable mechanisms of *N. sativa* involving the gene expressions of factors related to obesity and its pathogenesis have not yet been investigated in human studies.

Few clinical trials assessed the effects of *N. sativa* on serum concentrations of inflammatory markers and adipokines [18, 28–30]. Mahdavi et al. investigated the effects of *N. sativa* oil supplementation combined with a low-calorie diet on TNF-α [30] using a parallel-randomized design and reported that *N. sativa* oil may help to better manage weight and inflammatory status in obese women [28].

To the best of our knowledge, there are no clinical trials that assessed the effects of *N. sativa* or Thymoquinone through the TNF-α, PPAR-γ, or adiponectin pathways in obesity.

Consequently, the present clinical trial aimed to investigate the following objectives:

1. To assess the effects of oral *N. sativa* oil on the gene expression of TNF-α, PPAR-γ, and adiponectin along with the changes in serum concentrations of these factors among obese and overweight women.
2. To conduct a crossover, placebo-controlled, randomized clinical trial (RCT), to compare the altered gene expression as well as other outcome measures of every individual with herself, as her own control, and with the control group to achieve more accurate results.
3. To investigate the alterations in serum levels of liver enzymes including alanine transaminase (ALT), aspartate transaminase (AST), and alkaline phosphatase (ALP) as well as measuring lipid profile including total cholesterol, triglyceride, high density of lipoprotein cholesterol (HDL-C), low density of lipoprotein cholesterol (LDL-C), and atherogenic indices before and after every intervention period.

## Materials and methods

### Study design

**Fig. 1:**
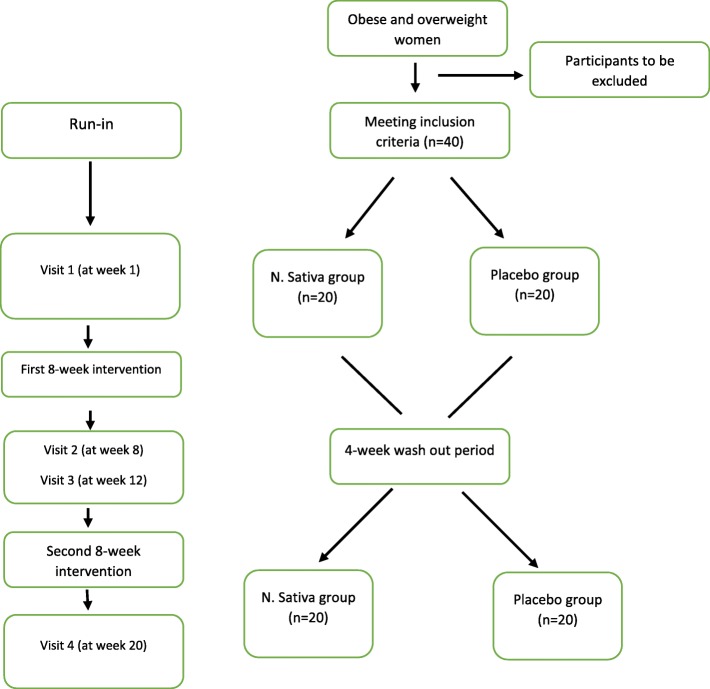
The overview of the study

**Fig. 2:**
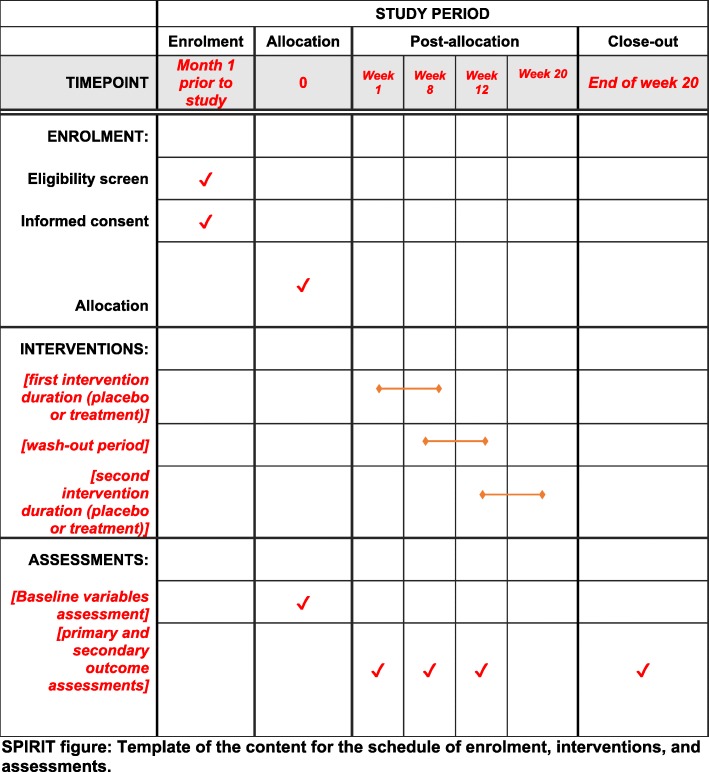
SPIRIT figure: Template of the content for the schedule of enrolment, interventions, and assessments

The design of the present study is a crossover, double-blind, placebo-controlled RCT which will be conducted among obese and overweight women for 20 weeks. Participants will receive either a daily dose of *N. sativa* or placebo for eight weeks in two phases separated by a wash-out period of four weeks. Each participant will be reversed for the second phase of the intervention according to the randomized crossover design. This study will be conducted in the Obesity Clinic of Shahid Sadoughi University of Medical Sciences, Yazd, Iran. The overview of the study is presented in Fig. 1. Moreover, this article will be reported according to the Standard Protocol Items: Recommendations for Interventional Trials (SPIRIT) statement [31]. A SPIRIT diagram detailing the timing of enrolment, interventions and assessments is provided in Fig. 2. A completed SPIRIT checklist is also provided in Additional file 1. Any methodological changes in the study design or sample size, which may potentially affect the participants’ safety or study procedures, will be discussed in the committee of ethics before the study initiation.

### Eligibility of criteria

Obese and overweight women aged 25–55 years with a body mass index (BMI) of 25–35 kg/m^2^ will be recruited from the Obesity Clinic in Shahid Sadoughi University of Medical Sciences. Exclusion criteria will include: cardiovascular diseases; hepatic, pancreatic, thyroid, or renal problems; having allergies to *N. sativa*; or a history of having a weight-reduction diet or program in the six months before the study. Furthermore, taking any anti-obesity medications or any herbal and anticoagulant agents including aspirin, vitamin E, vitamin K, and warfarin in the six months before the study as well as being either pregnant, lactating, or having uncontrolled polycystic ovary syndrome will also be considered exclusion criteria.

### Randomization and blinding

The present crossover, double-blind, placebo-controlled RCT will be consisted of two intervention periods of eight weeks each. Participants will be randomized 1:1 using stratified block randomization based on age (25–40 years and 40–55 years). One of the main researchers will use computer-generated random numbers to randomly allocate eligible participants into the intervention or control groups using sealed envelopes. Participants and administrators will be blinded to the content of the bottles, study treatment, and allocation until the final analysis. Individuals will be asked about their treatment group at the end of each intervention period. Their prediction will then be compared with the true assignments. Participants will receive four bottles for the entire 20-week intervention. Each bottle will contain 56 capsules and every participant will receive two bottles during every intervention period (one bottle at the beginning of week 1 and one bottle at the end of week 4 of every intervention duration).

Participants will be also asked about any possible reactions or different feeling after receiving the treatments at the end of every intervention period. No forms will be recorded, since the treatment doses were regarded as safe in the previous investigations.

### Compliance

Compliance with consumption will be monitored every other day through phone interviews which will be followed by face-to-face interviews once a week. Participants will be asked to consume one capsule before lunch and one capsule before dinner; this will also increase their compliance with consumption. All participants will be requested to bring back their first bottle at the end of the primer intervention and then they will be given the second bottle of supplements. If the remaining capsules of every participant will exceed 10% of the total administered supplements (12 capsules), the participant will be categorized as non-adherent. All individuals, including those who will complete the study or those who will not complete for any reasons, will follow the same schedule.

### Sample size

**Fig. 3:**
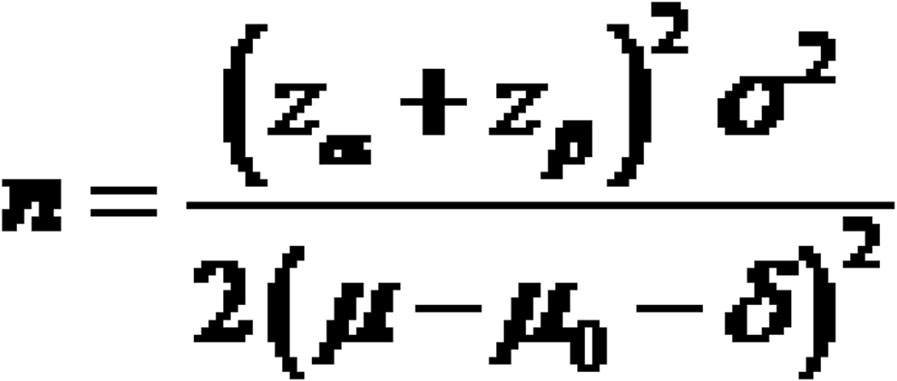
Formula for calculating sample size in crossover studies. δ is the clinically meaningful difference, μ2–μ1 is an expected true difference between the means, σ^2^ is the population variance, α is the significance level, and *β* is the power of the test

On the basis of sample size formula suggested for a balanced, crossover designed trials (Fig. 3), considering the non-inferiority margin of δ = 0.05 (clinically meaningful difference), with an expected true difference of 0.02 between the means, and a population variance of 0.01 and considering the significance level of α = 0.05, the sample size to achieve 80% power is calculated to be 35. The final sample size was increased to 40 to accommodate the expected 10% of dropout rate.

### Intervention

Before the study initiation, two trained researchers will introduce the study to participants. Participants, who will decide whether to join the investigation, will be asked to sign informed consent forms. The questionnaires will be reviewed and approved by the ethical committee of Shahid Sadoughi University of Medical Sciences. Data will be gathered by one of the main investigators. Individuals in the intervention group will receive two capsules of *N. sativa* (produced by Barij Essence Pharmaceutical Co.) and individuals in the placebo group will receive two capsules of paraffin oil per day. Both *N. sativa* and placebo capsules and the packs will be identical in terms of appearance, weight, texture, size, and smell and will only be differentiated by a code name (“A” or “B”). A person who is not involved in this project will label the containers “A” or “B.” Every capsule of *N. sativa* includes 1000 mg of *N. sativa* oil, which will consist of 0.01 mg of Thymoquinone-active component. The gas chromatography–mass spectrometry (GC/MS) test of every 50 g of *N. sativa* powder is presented in Table 1.

**Table 1:** Fatty acid content in every 50 g of *N. sativa* (GC/MS^a^ test)

Test	Reference method	Acceptable value	Result
Appearance	Visual	Brown color	Brown color
Odor	Organoleptic	Specific	Specific
Palmitic acid (%)	WR10^b^	11–14	11.84
Stearic acid (%)	WR10	–	2.00
Oleic acid (%)	WR10	17–26	22.14
Linoleic acid (%)	WR10	52067	59.66
Linolenic acid (%)	WR10	–	1.79
Thymoquinone (%)	EP^c^ 0.8 (2.2.23)	–	0.54

^a^Gas chromatography–mass spectrometry

^b^Rectangular waveguide size

^c^European Pharmacopoeia

### Matching energy intakes between groups

With regard to the dietary energy intakes of participants, we decided to give every individual an iso-energy dietary program in order to match every participant based on their energy and macronutrient intakes. Hence, an eight-week one-to-one individualized iso-energy dietary program for every intervention period, based on every participant’s current weight, will be performed. After determination of required energy, the registered dietician will develop a plan of servings from each food group that will fulfill the macronutrient distribution. The required participants’ daily energy intake will be distributed as follows: < 30% from fat (mainly mono-unsaturated and poly-unsaturated fatty acids); 55% from carbohydrates; and 1.2–1.5 g protein per kg of body weight. After baseline measurements, each participant will meet the registered dietitian three times during each intervention period (at weeks 0, 4, and 8).

Dietary intake will be quantified using three-day food records, including one weekend day and two weekdays as well as 24-h food recalls. Daily macro- and micro-nutrient intakes will be calculated by analyzing food data using nutritionist IV software (First Databank, San Bruno, CA, USA) modified for Iranian foods.

Shahid Sadoughi University of Medical Sciences will be responsible for any related problems; adverse events will be reported to the medical ethics committee from the initiation of the study. Participants may withdraw from the study for any reason at any time.

General information including age, diseases, medications, and supplements will be recorded through interviews at the beginning of the study. Physical activity level will be calculated through a questionnaire using metabolic equivalents (METs) at the beginning and at the end of every intervention period. Results of the assessments will be shared for every participant separately.

Primary outcomes consist of gene expression and serum concentration levels of adiponectin, PPAR-γ, and TNF-α. Secondary outcome measures consist of blood levels of lipid profiles and anthropometric measurements. The assessments of the outcomes are listed below.

### Gene expression assay

A total of 2 mL of fresh blood will be transferred into a tube consisting of ethylenediaminetetraacetic acid (EDTA); immediately after, peripheral blood mononuclear cells (PBMCs) will be isolated using density centrifugation with Ficoll-Paque [32]. Total mRNA will then be extracted using a related RNA kit [33]. Total extracted RNA will be then reverse transcribed to complementary DNA (cDNA) using a cDNA synthesis kit [34]. The mRNA gene expression of adiponectin, PPAR-γ, and TNF-α will be applied using the real-time polymerase chain reaction (PCR) method, after designing primers. The housekeeping gene will be glyceraldehyde phosphate dehydrogenase (GAPDH) in real-time PCR assessments.

### Biochemical measurements

A total of 12 mL fasting blood sample of venous blood will be collected from every participant. Circulating adiponectin and TNF-α will be assessed by enzyme-linked immunosorbent assay (ELISA). Blood pressure will be measured with a well-validated automated digital blood pressure monitor (Beurer, Germany, BM 85), following American Heart Association guidelines. Biochemical analyses including total cholesterol, triglycerides, HDL-C, LDL-C as well as liver enzymes, including ALT, AST, and ALP, will be measured using automated enzymatic methods. The values of atherogenic factors, including the ratios of HDL-C/LDL-C and LDL-C/HDL-C, will be calculated. All laboratory data will be identified by an ID number to maintain the participants’ confidentiality.

### Anthropometric measurements

The following measures will be quantified at the onset and at the end of every intervention period. Actual body weight, visceral fat, body fat, and body muscle percentage will be measured using a commercial body composition monitor and scale (InBody, USA, NO. 770). Body weights will be quantified to the nearest 0.1 kg. Measurements will be taken when participants are without shoes and wearing only light clothing. Their height will be measured to the nearest 0.1 cm with the participants barefoot using a stadiometer (Seca 222). BMI will be calculated as weight in kilograms divided by height in meters squared. Waist circumference measurement will be taken with minimal inspiration at the smallest waist circumference area, rounding up to the nearest 0.1 cm. Waist-to-hip ratio (WHR) will be calculated via standard equations.

### Statistical analysis

All statistical analyses will be performed with the Statistical Package for the Social Sciences (SPSS) (IBM SPSS Statistics for Windows, version 23.0; IBM Corp.), with significance set at *P* < 0.05. Normality of data will be assessed using the Shapiro–Wilk test. The values of continuous variables will be presented as means ± SEM. Categorical data will be presented as number and percentage in study groups. The general linear model repeated measure procedure will be incorporated to compare the quantitative outcome variables between the intervention periods in crude and multivariable adjusted models taking the rolling method and participants’ age, baseline values as between-person factors, and the change in physical activity level, energy intake, and body weight as covariates. The criterion for statistical significance will be defined as *P* < 0.05. Pearson’s correlation coefficient will be applied to show the correlation between biochemical measures and anthropometric indices.

We will analyze the results with intention to treat (ITT) and without participants with non-compliance (study completers). ITT analysis is based on the initial treatment assignment and not on the treatment eventually received. In the ITT method, participant-loss associated bias as well as misallocation or non-adherence of participants is near completely limited. In the case that researchers observe a significant difference between those allocated to receive an intervention and those who actually received that intervention (and adhere to it), per-protocol analysis, which is an additional analysis adjusted for actual treatment, will be performed. The results of the two mentioned analyses will be compared with each other.

## Discussion

The epidemic of overweight and obesity presents a major challenge to health around the world.

Adipose tissue predisposes pro-inflammatory status; hence, performing strategies to decrease body weight and the inflammation status would be of interest. *N. sativa* has recently been found to have anti-obesity effects, probably due to anti-inflammatory properties. However, the cellular pathways and mechanisms are not clear. Therefore, the present study will assess the effects of *N. sativa* on the gene expression of some factors involved in adipogenesis and obesity-related inflammation. If the results of the present study are valid, this will broaden the present assumptions on the specific functions of *N. sativa* and the context for performing more trials.

### Trial status

The initial participant recruitment has been completed.

## Additional file

Additional file 1: SPIRIT 2013 Checklist: Recommended items to address in a clinical trial protocol and related documents*. (DOC 132 kb)

## Data Availability

The datasets generated during the current study will be available via the corresponding author on reasonable request.

## Abbreviations

ALP: alkaline-phosphatase
ALT: alanine transaminase
AST: aspartate transaminase
BMI: body mass index
EDTA: ethylenediaminetetraacetic acid
ELISA: enzyme-linked immunosorbent assay
GAPDH: glyceraldehyde phosphate dehydrogenase
HDL-C: high density of lipoprotein cholesterol
ITT: intention to treat
LDL-C: low-density of lipoprotein cholesterol
METs: metabolic equivalents
*N. sativa*: *Nigella sativa*
PBMC: *peripheral blood mononuclear cell*
PCR: Polymerase Chain Reaction
PPAR-γ: peroxisome proliferator activated receptor alpha
TNF-α: tumor necrosis factor alpha
WHR: waist to hip ratio

## References

[1] Apovian CM (2016). Obesity: definition, comorbidities, causes, and burden. Am J Manag Care.

[2] Ellulu MS, Patimah I, Khaza'ai H, Rahmat A, Abed Y (2017). Obesity and inflammation: the linking mechanism and the complications. Arch Med Sci.

[3] Nigro E, Scudiero O, Monaco ML, Palmieri A, Mazzarella G, Costagliola C (2014). New insight into adiponectin role in obesity and obesity-related diseases. Biomed Res Int.

[4] Ahn J, Lee H, Kim S, Ha T (2007). Resveratrol inhibits TNF-alpha-induced changes of adipokines in 3T3-L1 adipocytes. Biochem Biophys Res Commun.

[5] Park PH, Huang H, McMullen MR, Mandal P, Sun L, Nagy LE (2008). Suppression of lipopolysaccharide-stimulated tumor necrosis factor-alpha production by adiponectin is mediated by transcriptional and post-transcriptional mechanisms. J Biol Chem.

[6] Motawi TK, Shaker OG, Ismail MF, Sayed NH (2017). Peroxisome proliferator-activated receptor gamma in obesity and colorectal cancer: the role of epigenetics. Sci Rep.

[7] Martin H (2010). Role of PPAR-gamma in inflammation. Prospects for therapeutic intervention by food components. Mutat Res.

[8] Diradourian C, Girard J, Pegorier JP (2005). Phosphorylation of PPARs: from molecular characterization to physiological relevance. Biochimie..

[9] Yadav A, Kataria MA, Saini V, Yadav A (2013). Role of leptin and adiponectin in insulin resistance. Clin Chim Acta.

[10] Mohammadi A, Gholamhoseinian A, Fallah H (2014). Zataria multiflora increases insulin sensitivity and PPARgamma gene expression in high fructose fed insulin resistant rats. Iran J Basic Med Sci.

[11] Zahorska-Markiewicz B, Janowska J, Olszanecka-Glinianowicz M, Zurakowski A (2000). Serum concentrations of TNF-α and soluble TNF-α receptors in obesity. Int J Obes.

[12] Stanley TL, Zanni MV, Johnsen S, Rasheed S, Makimura H, Lee H (2011). TNF-alpha antagonism with etanercept decreases glucose and increases the proportion of high molecular weight adiponectin in obese subjects with features of the metabolic syndrome. J Clin Endocrinol Metab.

[13] Ye J (2008). Regulation of PPARgamma function by TNF-alpha. Biochem Biophys Res Commun.

[14] Lucas S, Verwaerde C, Wolowczuk I (2009). Is the adipose tissue the key road to inflammation?. Immunol Immunogenetics Insights.

[15] Whitehead JP, Richards AA, Hickman IJ, Macdonald GA, Prins JB (2006). Adiponectin--a key adipokine in the metabolic syndrome. Diabetes Obes Metab.

[16] Koithan M, Niemeyer K (2010). Using herbal remedies to maintain optimal weight. J Nurse Pract.

[17] Namazi N, Larijani B, Ayati MH, Abdollahi M (2018). The effects of Nigella sativa L. on obesity: A systematic review and meta-analysis. J Ethnopharmacol.

[18] Datau EA, Wardhana, Surachmanto EE, Pandelaki K, Langi JA, Fias (2010). Efficacy of Nigella sativa on serum free testosterone and metabolic disturbances in central obese male. Acta Med Indones.

[19] Benhaddou-Andaloussi A, Martineau LC, Vallerand D, Haddad Y, Afshar A, Settaf A (2010). Multiple molecular targets underlie the antidiabetic effect of Nigella sativa seed extract in skeletal muscle, adipocyte and liver cells. Diabetes Obes Metab.

[20] Chehl N, Chipitsyna G, Gong Q, Yeo CJ, Arafat HA (2009). Anti-inflammatory effects of the Nigella sativa seed extract, thymoquinone, in pancreatic cancer cells. HPB.

[21] Abd-Elbaset M, Arafa EA, El Sherbiny GA, Abdel-Bakky MS, Elgendy AN (2017). Thymoquinone mitigate ischemia-reperfusion-induced liver injury in rats: a pivotal role of nitric oxide signaling pathway. Naunyn Schmiedebergs Arch Pharmacol.

[22] Zhu WQ, Wang J, Guo XF, Liu Z, Dong WG (2016). Thymoquinone inhibits proliferation in gastric cancer via the STAT3 pathway in vivo and in vitro. World J Gastroenterol.

[23] Park EJ, Chauhan AK, Min KJ, Park DC, Kwon TK (2016). Thymoquinone induces apoptosis through downregulation of c-FLIP and Bcl-2 in renal carcinoma Caki cells. Oxidative Med Cell Longev.

[24] Umar S, Hedaya O, Singh AK, Ahmed S (2015). Thymoquinone inhibits TNF-alpha-induced inflammation and cell adhesion in rheumatoid arthritis synovial fibroblasts by ASK1 regulation. Toxicol Appl Pharmacol.

[25] Benhaddou-Andaloussi A, Martineau L, Vuong T, Meddah B, Madiraju P, Settaf A (2011). The in vivo antidiabetic activity of nigella sativa is mediated through activation of the AMPK pathway and increased muscle Glut4 content. Evid Based Complement Alternat Med.

[26] Pei X, Li XL, Chen HM, Han Y, Fan YG (2016). Thymoquinone inhibits angiotensin II-induced proliferation and migration of vascular smooth muscle cells through the AMPK/PPAIR gamma/PGC-1 alpha pathway. DNA Cell Biol.

[27] Awad ASM, Abd Al Haleem EN, El-Bakly WM, Sherief MA (2016). Thymoquinone alleviates nonalcoholic fatty liver disease in rats via suppression of oxidative stress, inflammation, apoptosis. Naunyn Schmiedebergs Arch Pharmacol.

[28] Mahdavi R, Alizadeh M, Namazi N, Farajnia S (2016). Changes of body composition and circulating adipokines in response to Nigella sativa oil with a calorie restricted diet in obese women. Journal of Herbal Medicine.

[29] Mahdavi R, Namazi N, Alizadeh M, Farajnia S (2015). Effects of Nigella sativa oil with a low-calorie diet on cardiometabolic risk factors in obese women: a randomized controlled clinical trial. Food Funct.

[30] Mahdavi R, Namazi N, Alizadeh M, Farajnia S (2016). Nigella sativa oil with a calorie-restricted diet can improve biomarkers of systemic inflammation in obese women: A randomized double-blind, placebo-controlled clinical trial. Andrologia..

[31] Chan A.-W., Tetzlaff J. M., Gotzsche P. C., Altman D. G., Mann H., Berlin J. A., Dickersin K., Hrobjartsson A., Schulz K. F., Parulekar W. R., Krleza-Jeric K., Laupacis A., Moher D. (2013). SPIRIT 2013 explanation and elaboration: guidance for protocols of clinical trials. BMJ.

[32] Loos J.A., Roos D. (1974). Ficoll-isopaque gradients for the determination of density distributions of human blood lymphocytes and other reticulo-endothelial cells. Experimental Cell Research.

[33] QIAzol Lysis Reagent, catalog number: 79306. [http://www.labsave.com/products/qiazol-lysis-reagent-200ml-79306--6] Accessed Jan 2009.

[34] cDNA Synthesis Kits—Thermo Scientific | Thermo Fisher Scientific - TR. [https://www.thermofisher.com/order/catalog/product/K1622].

